# Time constraints may pace the ontogeny of movement behaviour

**DOI:** 10.1098/rspb.2022.2429

**Published:** 2023-04-12

**Authors:** Anne G. Hertel, Ron Efrat, Korin Reznikov, Nir Sapir, Oded Berger-Tal, Thomas Mueller

**Affiliations:** ^1^ Behavioural Ecology, Department of Biology, Ludwig-Maximilians University of Munich, Planegg-Martinsried 82152, Germany; ^2^ Senckenberg Biodiversity and Climate Research Centre (SBiK-F), Frankfurt (Main), Hessen, Germany; ^3^ Mitrani Department of Desert Ecology, Jacob Blaustein Institutes for Desert Research, Ben-Gurion University of the Negev, Midreshet Ben-Gurion 8499000, Israel; ^4^ Department of Evolutionary and Environmental Biology and Institute of Evolution, University of Haifa, 3498838 Haifa, Israel; ^5^ Department of Biological Sciences, Goethe University Frankfurt, Max-von-Laue-Straße 9, 60438 Frankfurt (Main), Germany

**Keywords:** egyptian vulture, locomotor performance, behavioural development, migration, soaring flight

## Abstract

During early development, juvenile animals need to acquire a diverse behavioural repertoire to interact with their environment. The ontogeny of animal behaviour, is paced by the motivation to improve, e.g. internal clocks, and limited by external constraints, e.g. weather conditions. We here evaluate how naive Egyptian vultures (*Neophron percnopterus*) improve in locomotor performance, measured as daily maximum displacement, prior to their first migration under three different time constraint regimes: we compared wild hatched vultures, migrating one month after fledging, with captive-hatched vultures, released in spring four months or in winter nine months before migration. We found that the time until migration paced the development of movement behaviour: wild birds rapidly increased displacement distances within the first two weeks after fledging, while spring and winter released vultures delayed movement increases by two and four months, respectively. Under relaxed time constraints captive-hatched vultures displayed diverse functional forms of performance enhancements and therefore great variability in individual ontogeny of movement behaviour. While weather conditions in winter could limit flight movements, some birds indeed moved immediately after their release, indicating that weather may not be limiting. Our findings promote the idea that relaxed ecological constraints could uncover hidden phenotypic flexibility in ontogeny, which could present a greater potential for adaptability under environmental change than currently expected.

## 1. Introduction

Animals flexibly adjust their behaviour or acquire new behaviours to cope with prevailing and changing environmental conditions [1,2]. Juveniles in particular need to acquire and master a set of specific behaviours to successfully interact with their biotic and abiotic environment, enabling them to find resources, secure survival and ultimately to reproduce. The ontogeny of a single behaviour describes the enhancement in performance from a naive, untrained state to a trained state [3]. This process can be innate (i.e. genetically determined), or acquired through social or individual learning [4,5]. In addition, environmental constraints are expected to moderate the timing and pace of performance enhancement in juvenile or naive animals [6]. Specifically, animals facing severe stress or strict constraints, such as high predation pressure, need to improve their performance more rapidly than animals facing more relaxed conditions [7]. Moreover, under relaxed constraints, we expect the emergence of individual variation in performance and specifically high between-individual variability in the rate of performance enhancement [8]. This prediction follows a more general hypothesis that environmental constraints limit phenotypic variability. For example, in the absence of selective constraints in the form of predation or food limitations, populations express greater among-individual variability than under heightened predation pressure or food stress [9–11]. Additionally, environmental conditions that are rarely encountered by a population may allow for increased phenotypic variation because no past selection has been asserted under these novel conditions [12]. Yet, it is still unknown to what extent environmental constraints indeed determine the ontogeny of behaviour in the wild and whether predictions of increased phenotypic variability under relaxed constraints also hold true for behavioural ontogeny.

**Figure 1.**
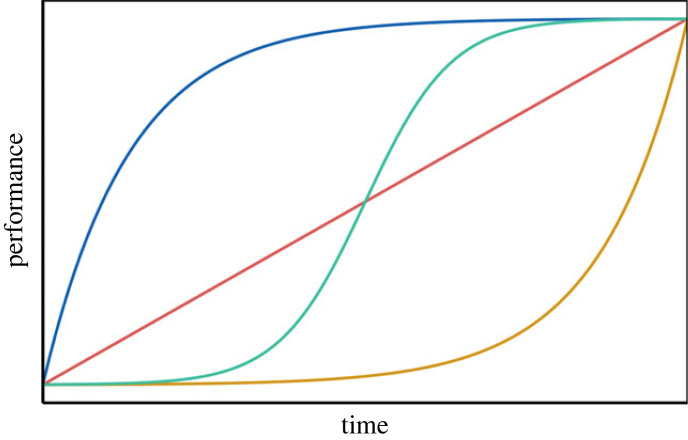
Potential relationships between elapsed time and increases in performance. Learning with experience can take different shapes: linear (red), asymptotic (blue), exponential growth (yellow) or sigmoidal (green).

To capture the full ontogeny of a behaviour we need to repeatedly assess behaviour over discrete time steps, starting with its first occurrence. Movement data can provide a unique opportunity to record spatial behaviours continuously over time from the first steps, through immaturity into adulthood [4,13,14]. Thereby, enhancement in performance over time can be approximated using simple mathematical functions. In experimental behavioural biology, performance curves (also referred to as learning curves) have been used to describe task-performance increases in laboratory-based operant conditioning paradigms [15,16]. We can distinguish four primary pathways: *linear* (figure 1, red line), a consistent performance enhancement over time; *asymptotic*, an exponential rise to limit (figure 1, blue line) where improvement is rapid in the beginning but slows over time and eventually plateaus [17]; *exponential* (figure 1, yellow line), a slow rate of improvement which accelerates over time; and *sigmoidal* (figure 1, green line), performance enhances slowly in the beginning, followed by a period of rapid performance increase up to a limit, where performance plateaus and shows no further improvement [15]. Different functional forms of performance curves reflect, for example, whether an easy or complicated task is being learned, where the latter requires more repetitions to reach proficiency. While on the population level, performance curves often take a gradually increasing shape, substantial among-individual variation in learning speed and terminal performance ability have been documented [6,15,18]. However, whether individuals of the same species can respond to a common problem with different functional forms of performance enhancements has not been investigated yet.

Here we use a unique dataset of individual-based daily flight movements of juvenile Egyptian vultures (*Neophron percnopterus*) collected from fledging until the start of their first migration to study the development of movement behaviour prior to first migration. Specifically, we compared three groups of vultures that differed in the time constraints between fledging and migration, the associated prevailing weather conditions, and rearing type. We were specifically interested in whether vultures with more time between fledging and migration would show increased phenotypic variability in their ontogeny of movement. Environmental cues such as photoperiod, so-called zeitgebers, synchronize the bird's internal clock to the annual rhythm and indicate, for example, the timing of migration [19]. For juvenile animals, the onset of migration represents a major target for the development of movement behaviour: long before the actual onset of migration, juveniles need to start improving their movement capacity in order to reach a movement performance that is sufficient to master the long daily distances characteristic for migration [20,21]. The time between a juveniles first movement and the start of its first migration, i.e. the time constraint to master movement, can vary tremendously from days to years, even within species [22]. In many avian species, the first migration occurs within weeks after fledging and becoming independent [23]. For migratory bird species, first-time migration is the period in which most mortality occurs [24–26] and higher pre-migratory flight experience has been associated with increased survival [27] suggesting a strong selective pressure for birds to start flying as early as possible. We, therefore, expect environmental cues related to migration to act as internal clock (zeitgeber), which triggers the motivation to move. Hence, we expect the time between fledging and migration onset to pace the ontogeny of movement behaviour.

The Egyptian vulture's primary movement mode is soaring-gliding flight, where birds exploit air updrafts (thermals) created by the heating of the ground to gain height at low metabolic cost [28–30]. Soaring enables them to cover daily distances of 140–300 km during migration [31] and routine daily movement distances of sub-adult Egyptian vultures (i.e. individuals which have completed one migration cycle) in their summer range are around 30–35 km (electronic supplementary material, analysis S3). Best conditions for soaring flights occur in the hot dry summer months while thermals are less strong in the cooler, rainy winter months, limiting the birds' capacity to move over long distances. Previous studies suggest that this flight mode requires a longer learning period [32]. For example, red kites (*Milvus milvus*) initially use an active, flapping flight mode and only over time acquire soaring-gliding flight [20], and juvenile Eurasian griffon vultures’ (*Gyps fulvus*) flight efficiency is lower than that of adults, in particular under challenging wind conditions [33].

**Figure 2.**
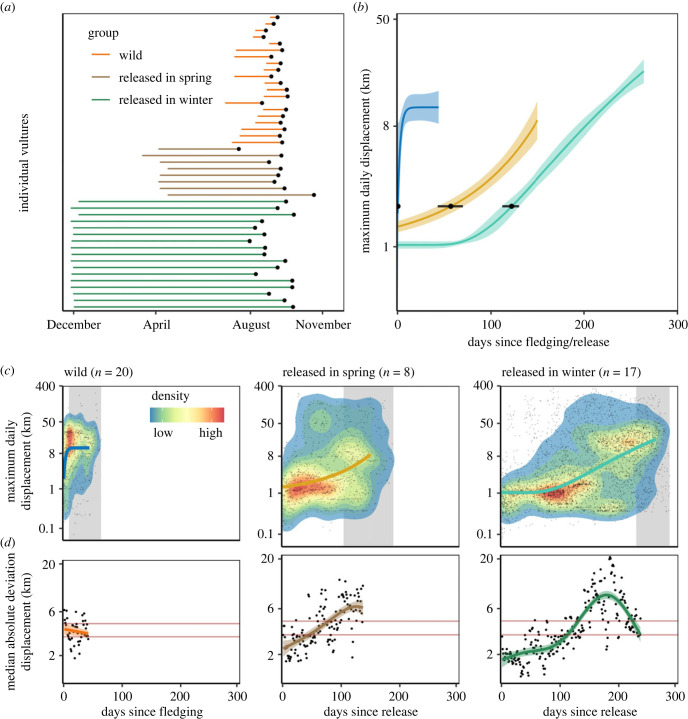
We obtained tracking data of 20 wild hatched and 25 captive hatched Egyptian vultures (*Neophron percnopterus*) and quantified maximum daily displacement between fledging/release and the start of migration. (*a*) Wild hatched vultures (orange) fledged between July and August and departed on their first migration between August and September. Captive hatched vultures were released in spring (brown) or winter (green) and departed on their first migration 4 or 9 months after their release. (*b*,*c*) At the median group level, wild vultures showed an asymptotic increase in displacement distance between fledging and departure on migration (grey area showing range of migration departure dates) while spring and winter released vultures delayed initial increases in movement and showed exponential and sigmoidal performance increases, respectively. (*d*) The median absolute deviation in displacement distance over a three-day moving window (km) was higher in the spring and winter released birds, especially with increasing time since release, compared to the wild hatched birds. Models were fitted on log transformed maximum daily displacement distances and hence *y*-axes are on the log scale, while labels are shown in kilometre units to aid interpretation. Dots in panel (*c*) are showing the raw daily displacement data, dots in panel (*d*) represent the median absolute deviation in displacement over a three-day moving window, the heatmap in panel (*c*) shows the density distribution of the raw data from low density (blue) to clustered high density (red), lines (*b,c,d*) are showing model predictions with 95% confidence intervals (*b,d*).

We evaluated how flight performance, measured as maximum displacement from the daily roost, increased over time, from fledging to the start of the first migration in three groups of Egyptian vultures (figure 2*a*): (1) wild hatched juveniles fledging in summer approximately 1 month before migration; (2) vultures hatched in captivity and then released into the wild in spring, 4 months prior to migration; and (3) vultures hatched in captivity and released into the wild in winter, 9 months prior to migration. We expected that the three groups differ in their ontogeny of movement behaviour due to systematic differences in the time constraints imposed on them by the timing of migration. However, we acknowledge that time constraints potentially are confounded with weather and rearing conditions. Birds released in winter experience less ideal flight conditions (fewer thermals) during early ontogeny, potentially constraining their capacity to move while birds released in spring and wild hatched birds meet better weather conditions during early ontogeny. In addition, systematic differences imposed by different rearing conditions, e.g. no parental learning of flight in captive reared birds but a release in cohorts of similarly inexperienced birds at a supplemental feeding site, and different maturation state due to a different age of wild fledged birds and released birds, could confound our conclusions regarding time constraints. We discuss the merit of each of these mutually non-exclusive drivers of movement ontogeny based on a series of population- and individual level analyses. If time constraints affect movement ontogeny, we expect that on the population level, wild birds hatching 1 month before migration departure, will show immediate and rapid increases in movement distances resembling an asymptotic performance curve while birds released 4 and particularly 9 months before migration would delay initial increases in movement. Because we assume that time to migration drives ontogeny of movement, we expected that birds released 9 months before migration departure, would delay increases in movement for longer than birds released 4 months before migration departure. To that end we expected systematic differences in movement distance between wild-hatched and captive-hatched Egyptian vultures in the days immediately after fledging/release but not in the days prior to migration. We further expected that the relaxed time constraints experienced by captive-hatched individuals allow for individual variation in movement ontogeny to emerge: We expected this to manifest in (a) greater variability in the two captive groups as compared to the wild group at the population level, and (b) greater individual level variation in both shape of performance curves (figure 1) as well as time until increase of movement distances. We tested for maturation, parental and social effects on the shape of performance enhancement and latency to reach the wild birds' displacement distance.

## 2. Methods

### (a) Data collection

Captive-hatched Egyptian vultures were raised in Hai-Bar Carmel Nature Reserve breeding facilities in northern Israel (32.75° N, 35.01° E) (see ref [34] for details regarding the captive-breeding and release methods). Hatched chicks were released to the wild between 2013 and 2020, in spring (March or April) or winter (December), during their second or third year of life. Location data from both captive-bred and wild-hatched vultures was collected by fitting the birds with either Argos-GPS transmitter (manufactured by Microwave Telemetry), or GPS-GSM transmitters (E-obs or Ornitela); all transmitters were solar-powered. Transmitters were fitted to captive-hatched vultures between two hours and two days prior to their release. Wild-hatched vultures were aged according to plumage characteristics and were tagged in the nest approximately 5–20 days before their expected fledging (ca. 75 days post hatching). Transmitters were fitted in either backpack or leg-loop harness configurations [35] and weighed less than 2% of the vultures’ mass [36]. GPS data were collected during daylight time, starting an hour before sunrise and ending an hour after sunset, a time period that incorporates all of the Egyptian vultures' movements. Sample rate varied from 1 Hz to 60 min, depending on tag settings and battery voltage.

### (b) Processing movement data

All GPS data were re-sampled to the coarsest sampling rate: a 60-minute location interval. Because wild-hatched vultures were tagged in their nests, where the solar panels are not exposed to the sun and thus the battery cannot be charged, few GPS locations were recorded during some post-fledging days. We only included days with at least 10 locations in our analysis. Furthermore, to avoid including data collected during pre-fledging days we defined fledging as the first day in which a vulture moved at least 200 m from its nest, as the spatial error of hatchlings in the nest was around 100 m. For each day, the maximum daily displacement from the roost was calculated as the furthest distance recorded from the first location of that day. Because the first GPS location was taken an hour before sunrise, we assumed that it represents the vultures’ night roost, thus maximum daily displacement represents the furthest daily exploration. We used the geosphere package [37] in the R environment [38] for distance calculations.

### (c) Statistical analysis

We log-transformed displacement distances for all statistical analyses. Log transformation was important to comply with statistical assumptions of normality but further underpinned our biological assumption that increases in movement at shorter distances are more likely to reflect performance increases than at longer distances. For biological interpretation we report results back-transformed to the km scale.

#### (i) Wild and captive performance curves and variability around those curves

We fitted population-level performance curves separately for wild birds and captive birds released in spring or winter. We fitted four competing functional relationships between movement distance (log transformed) and number of days since fledging or release: a linear relationship, an exponential growth model, an asymptotic growth model and a four-parameter Weibull function which produces a sigmoidal curve (figure 1). We additionally fitted a null model indicating no relationship between movement distance over days since release. We used the R packages *drc* [39] and *aomisc* [40] for self-starting nonlinear curve fitting. Because of highly scattered data we opted for more robust curve estimation on the median (as opposed to non-robust least-squares estimation on the mean). Accordingly, the linear model was estimated using quantile regression on the median using the R package ‘quantreg’ [41]. We compared model fit using Bayesian information criterion (BIC) where lower values of BIC indicate better model fit. When BIC values of several models were similar (within the range of two, indicating an equally good fit), we selected the simplest model. We preferred BIC over AIC because it penalizes additional model parameters more strongly and we were interested in the simplest curve describing the increase in displacement distance over day since fledging/release. We bootstrapped each group's selected model 1000 times to derive 95% confidence intervals for the predicted increase in movement along time. We determined the median distance covered by wild birds on their first day after fledging and extracted the number of days (±95% CI) after which the spring and wild birds' predicted movement distances had increased to 2 km, as indicator that vultures had started to increase movement performance. To assess the variability of displacement distances around the median we estimated the median absolute deviation for a three-day moving window. We tested for group-specific changes in variability along time since fledging/release by fitting group-specific temporal smoothers with 4 degrees of freedom using the ‘mgcv’ package. The results were qualitatively similar to non-overlapping daily estimates of the median absolute deviation but days with few datapoints were less influential.

#### (ii) Maximum displacement after fledging and before migration

We first fitted two mixed effects models to compare movement behaviour of wild and captive hatched birds, released either in winter or spring, in the first 20 days after fledging or release and the last 20 days before departure on migration. For wild birds, these two periods overlapped in most birds because wild birds only stayed on average 32 days in the area before migrating (range: 9–64; figure 2*a*). We only included birds in the comparison for which we obtained movement behaviour on at least 9 days of the 20-day periods, which reduced the samples size for the period 20 days after fledging by six wild birds (retaining 14 out of 20 birds) and four winter released birds (retaining 13 out of 17 birds). For both periods we fitted a model controlling for interactive effects of rearing type (wild, released in spring or released in winter) and time (i.e. days since fledging/release and days to migration) on log transformed maximum daily displacement. We allowed for a nonlinear increase (or decrease) in movement over time with a second order polynomial. Because we expected *a priori* that movement behaviour increases in a nonlinear fashion and that groups may differ in their movement after fledging and prior to migration, we fitted a full model and interpreted model coefficients rather than applying model selection. We controlled for among-individual variation in movement with random intercepts for individual identity and random slopes over time, grouped by rearing type and we also allowed the residual variance to vary between rearing types. Grouping variance components by rearing type was important as we expected group specific differences in among-individual and residual variance. We used the R package *brms* [42] to fit mixed-effects models and inspected posterior predictive plots for model fit. We ran four chains for 10 000 iterations with a warmup of 8000, resulting in 800 posterior draws.

#### (iii) Individual performance curves

For each individual we fitted five competing functional relationships between movement distance (log transformed) and number of days since release and selected the simplest model within a BIC range of two (see ‘wild and captive performance curves' for details). Individual models were fitted on the mean (least squares estimation) and not on the median because individual data distributions where symmetrical and with fewer outliers than the data distribution on the population level. Using each individual's top model, we calculated after how many days each bird reached the average movement distance of wild birds in their first 20 days after fledging. We tested whether wild, spring and winter released birds showed equal variance in reaching the benchmark using a Levene's test. We used a Kruskal Wallis ANOVA to test whether the median time to reach the benchmark flying distance differed between wild, spring and winter released birds. For captive reared birds we also tested whether age at release (maturation), release cohort (social environment), or parental breeding pair (genetic relatedness and other potential effects related to the specific pair such as parent age and behaviour) would explain similarities among birds in the type of performance curve, and the likelihood and time to reach a benchmark flying distance (see electronic supplementary material, analysis S1 for details).

## 3. Results

We obtained data from 20 wild-hatched and 25 captive-hatched Egyptian vultures (figure 2*a*). Wild birds hatched in May and fledged at an age of approximately 75 days. Captive hatched birds were raised and released as part of a conservation-translocation project [34]. These birds were the descendants of five captive breeding pairs and were released in eight cohorts between 2013 and 2020 either in spring (March–April, *n* = 8 birds) or winter (December, *n* = 17 birds) before their first migration in August–October (figure 2*a*). The age at release ranged from 155–701 days. The time between fledging/release and the onset of migration was on average 32 days (range: 9–64 days) for wild birds, 153 days (104–189 days) for birds released in spring and 261 days (230–287 days) for birds released in winter.

### (a) Performance curves and variability under strict and relaxed time constraints

We found variation in performance curves among wild hatched, spring released and winter released vultures. Increases in log transformed maximum daily displacement distance in wild vultures were best approximated by an asymptotic growth curve (electronic supplementary material, table S1). Wild vultures' median movement increased rapidly from around 2 km on the first day after fledging, reaching a plateau after about 6 days (s.e. = 1.6 days) (figure 2*b*,*c*). By contrast, spring and particularly winter released vultures delayed increases in movement and performance followed an exponential and sigmoidal curve, respectively (figure 2*b*,*c*). Spring released birds were predicted to increase movement at an initially slow rate, reaching a median maximum daily displacement of 2 km (the distance wild birds covered on their first day after fledging) only after 58 days (lower and upper confidence limit = 43, 70 days; approximately beginning of June, given a release in the beginning of April; figure 2*a*). Winter released vultures were predicted to stay sedentary for even longer and only moved over 2 km after 122 days [112, 130 days; approximately mid-April, given a release in the beginning of December] (figure 2*b*,*c*). Across wild birds we found a median absolute deviation in maximum daily displacement over a 3-day moving window of 1.3 km with no significant increase or decrease over time between fledging and the start of migration (lower and upper credible interval reflecting red lines in *d* = 1.17 km, 1.5 km). In comparison, individual variability in daily displacement (measured as median absolute deviation) of spring and winter released vultures immediately after their release was 0.8 km (credible interval = 0.7 km, 1 km) and 0.6 (0.4, 0.7) km, respectively, which was significantly lower than the individual variability of wild birds, as inferred by a lack of overlap of the groups’ credible intervals with the wild bird's median absolute deviation credible interval (red lines in figure 2*d*). However, variability of spring and winter released birds increased with time since release and peaked at 1.9 (1.7, 2) km and 2.1 (2, 2.2) km, 130 and 180 days after release, respectively, which was significantly higher than variability in wild vultures (no overlap of credible intervals with red lines in figure 2*d*). The higher median absolute deviation of spring and winter released vultures compared to wild birds suggests greater individual variability in movement distances.

### (b) Maximum daily displacement after fledging and before migration

**Figure 3.**
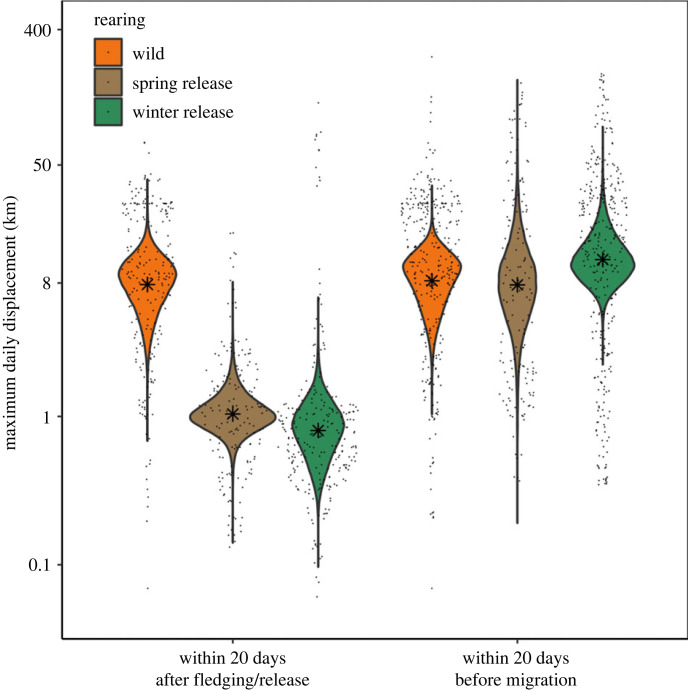
A linear mixed-effects model showed that wild birds moved over significantly farther distances in the first 20 days after fledging (mean = 8.1 km) in comparison to spring (mean = 1.0 km) or winter released captive birds (mean = 0.8 km). This discrepancy disappeared when comparing the three groups in the last 20 days before migration departure as wild hatched and both spring and winter released birds moved over similar distances per day. Models were fitted on log transformed maximum daily displacement distances and hence y axes are on the log scale, while labels are shown in kilometre units to aid interpretation. Dots are showing the raw maximum daily displacement data and violins are showing modelled posterior distributions with means indicated by asterisks.

Maximum daily displacement distance during the first 20 days after fledging was higher for wild birds than for winter or spring released vultures (electronic supplementary material, table S2). Our model results show that wild hatched vultures increased maximum displacement from an estimated 3.7 km to an estimated 8.2 km over the 20-day period (electronic supplementary material, figure S1A) and therefore moved on average 8.1 km [credible interval: 7.7 km, 8.5 km] per day (figure 3). Vultures raised in captivity did not increase movement between day 1 and day 20 after their release (electronic supplementary material, figure S1A) and moved over much shorter daily distances (spring released: 1.1 km [1.02 km, 1.09 km], winter released 0.85 km [0.8 km, 0.89 km]; figure 3). These differences between wild and released vultures disappeared when comparing movement in the 20 days before migration (electronic supplementary material, table S2): vultures of all three groups moved over similar average daily distances (wild = 8.7 km [4.5 km, 11.8 km]; spring released = 8.1 km [5.95 km, 9.28 km], winter released = 11.5 km [9.4 km, 17.6 km], figure 3; electronic supplementary material, figure S1A).

### (c) Individual variation in performance curves under relaxed time constraints

**Figure 4.**
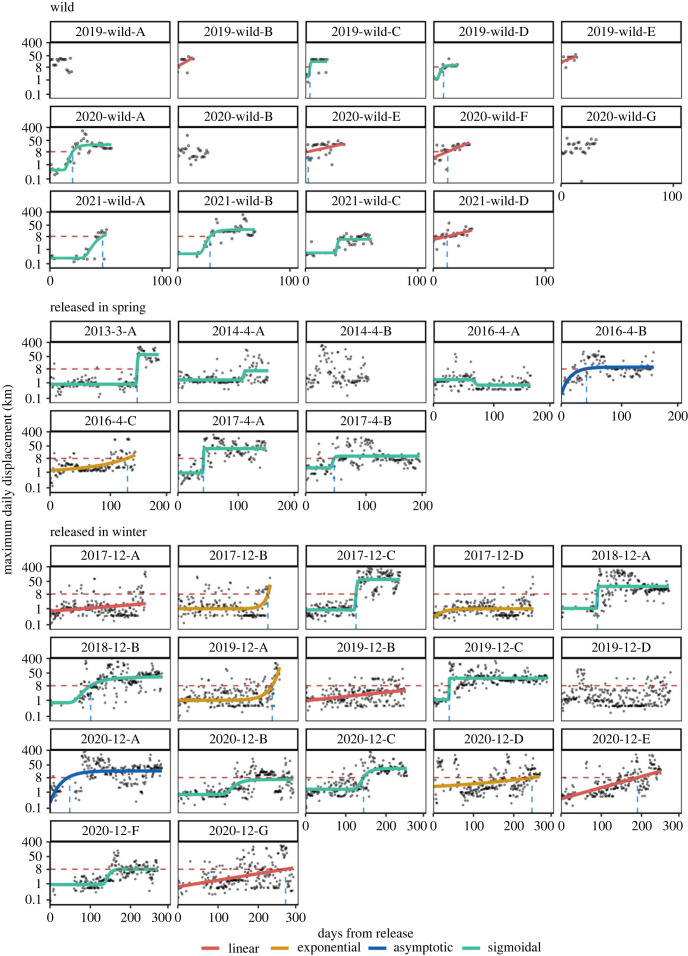
To study individual ontogeny of movement, measured as maximum daily displacement (km) in Egyptian vultures from fledging/release to the start of the first migration, we fitted for each individual five alternative performance curves of different functional forms: null, linear, asymptotic, exponential, sigmoidal. Using BIC to select the most parsimonious performance curve revealed different patterns for time constrained wild-hatched birds and captive-reared vultures released under relaxed time constraints. Performance in spring and winter released vultures followed a sigmoidal Weibull curve in 12 cases, for 4 birds the linear model reflected increase in movement distance best, for 5 birds the exponential and for 2 birds the asymptotic model performed best. Two birds showed no increase in movement distance. For wild hatched vultures, performance increased linearly in 5 cases, sigmoidally in 6 cases, and 3 birds showed no increase, yet moved around 8 km from the first day on. The red dotted line indicates the modelled average movement distance for wild birds in the first 20 days after fledging (8.1 km). The value on the *x*-axis where the red dashed line and an individual's performance curve cross indicates how many days it took for that individual to show a similar flight proficiency as an average wild hatched bird after fledging. Panels are labelled by release cohort s (release year-release month-letter identification) or the year a bird hatched and tagged. Models were fitted on log transformed maximum daily displacement distances and hence *y*-axes are on the log scale, while labels are shown in kilometre units to aid interpretation.

We found large individual variation in the timing and speed of movement increases (figure 4). Of 25 captive reared individuals, 12 displayed a performance increase best described by a sigmoidal Weibull function, where the bird first moved very little for a prolonged period of time, followed by an increase of daily movements up to a plateau where daily movement distances did not increase further. Four birds showed a consistent linear increase in movement distance, five an exponential increase immediately before migration, two an asymptotic increase with rapid increases in flight distance shortly after release and two birds showed no consistent increase in distance at all (electronic supplementary material, table S3). Of 14 wild birds, 5 showed a linear increase in movement and 6 showed a sigmoidal increase with an initial delay. Three birds showed no performance increase, however, their flight distance immediately was around or above 8 km. The age of the bird, cohort or breeding pair identity did not determine the type of performance curve in captive birds (electronic supplementary material, table S4, figure S3).

Individual performance curves revealed great variability in the time to reach a displacement distance of an average wild bird during their first 20 days after fledging (8.1 km, red dashed line in figure 4). While only one out of 14 wild birds did not reach the benchmark distance of 8.1 km, 7 of the 25 captive-raised birds never reached this distance. Wild birds took a median of 6 days after fledging to reach the benchmark (range 0–44 days after fledging, variance = 192, blue dashed lines in figure 3). By contrast spring released birds reached the 8.1 km after a median of 45 days (within a 121-day range, variance = 2593) and winter released birds reached the benchmark after a median of 144 days (within a 228-day range, variance = 6590). Both the variance (*F* = 9.97, *p* < 0.001) and median (*χ*^2^ = 20.1, *p* < 0.01) in the number of days to reach the benchmark differed significantly among wild, spring and winter released birds. Age at release, cohort or breeding pair identity neither affected the likelihood for captive birds to reach a daily displacement of 8.1 km prior to migration nor the number of days it took birds to reach it (electronic supplementary material, table S4). For example, out of seven birds released together, one bird never reached a daily distance of 8.1 km while the other birds reached that daily distance after 48, 144, 172, 188, 241 and 264 days respectively (cohort 2020-12 in figure 4).

## 4. Discussion

Taken together, our results suggest that migration phenology paces the ontogeny of movement behaviour and further, that relaxing time constraints imposed by migration departure date allows for variation in individual development to emerge. Movement performance of wild Egyptian vultures fledging 1 month before migration enhanced immediately after fledging. By contrast, vultures released in spring 4 months prior to migration remained almost stationary for the first two months and vultures released in winter 9 months prior to migration for almost four months after their release. However, individual ontogeny of movement varied greatly among released birds with a handful of vultures starting to move right away, similar to their wild counterparts, and others showing delayed performance enhancements to various degrees. A few individuals never increased movement until their departure for migration. While wild-hatched vultures also showed rudiments of different strategies, their strict time schedule restricted the opportunity for individual variability in ontogeny. This study is the first to demonstrate a possible effect of time constraints on the schedules of juvenile behavioural ontogeny in the wild.

Relaxed environmental constraints can lead to increased phenotypic variability [43], in the short-term through increased phenotypic plasticity [44,45] and in the long-term through relaxed natural selection and evolution [46]. While the effect of constraints on behavioural variability has been mainly studied in the context of predation (with relaxed predation pressure leading to increased behavioural variability in prey species) [9,11], relaxation of other environmental constraints can equally promote behavioural variability. Moreover, past studies almost exclusively focused on behavioural variability among adult individuals but to our knowledge this is the first study testing the effect of environmental constraints on individual variation in behavioural ontogeny. We here illustrate that under strict time constraints created by the onset of migration, individuals showed little variability in behavioural ontogeny, with the majority of birds moving over long distances within 20 days after fledging. By contrast, increasingly relaxed time constraints created ecological opportunity and led to pronounced variability in ontogeny. Time constraints were however confounded with weather conditions, where winter released vultures met worse flying conditions in early ontogeny than spring released and particularly wild hatched vultures. Yet, at least on the individual level, weather did not seem to be the main ecological constraint as several winter released vultures started flying before the arrival of warmer weather in spring. Our results suggest that flexibility in ontogeny is maintained and can be expressed when an ecological opportunity occurs.

Importantly, we show here that birds with relaxed time constraints pursued very different functional forms of performance enhancements, whereas in experimental conditioning paradigms individuals usually respond with a similar performance curve to a given learning problem and only differ in their timing to start improving or reaching a performance plateau [15]. It therefore remains the question which proximate factors could mediate the observed variability in group-level and individual ontogeny. In fact, the shape of the performance curve harbours important information about how physiology and motivation limit the ontogeny of behaviour. In the case of animal movement, these potential limitations mirror the mechanistic components driving animal movement described under the movement ecology paradigm [47], where differences in movement ontogeny could be explained by (a) differences in motion capacity (e.g. different ages or molt stages), (b) differences in the internal state (e.g. internal clock, hunger level) of an animal which determines its motivation to improve movement, and (c) differences in external factors (e.g. weather conditions) which can affect both the capacity and the motivation of an animal to improve movement [47]. Rapid initial increases, as we found for the majority of our wild-hatched and some of our captive-hatched birds indicate that physiology is not limiting and that motivation is high. By contrast, slow rates of initial improvement, as we found for most of our captive-hatched birds and particular ones released on the most relaxed time regime 9 months before their first migration, suggest that either physiology or external conditions (i.e. weather) are limiting or that there is a lack of initial motivation. All captive-hatched birds were reared under similar conditions in cages and the age of the bird did not explain individual differences in movement patterns. We therefore deem it unlikely that differences in motion capacity between wild and captive-hatched birds caused variability in movement ontogeny. Rather, captive-hatched vultures might be less motivated to start enhancing movement immediately, because they are released in the vicinity of supplemental feeding sites, because they are lacking the company of experienced adults, precluding the option for social learning of soaring flight behaviour [5], or because their internal clock, only triggers the motivation to start moving when migration approaches [19]. External weather conditions probably have additive effects on the motivation and capacity to move. For soaring-gliding flight, birds harness thermals, which are more common in the hot and rainless summer [29]. Because of the progressively delayed increase in movement ontogeny along progressively relaxed time constraints to migration from 1 to 4 to 9 months, we believe that an internal clock in combination with weather conditions explains the group-level variation in movement enhancement best. Additionally, because birds released 9 months prior to migration delayed movement for longer than spring-released birds we deem it unlikely that the observed pattern was solely driven by differences in rearing conditions (see also electronic supplementary material, analysis S2). Interestingly, birds within the three groups were exposed to the same conditions, yet displayed different degrees of individual variation in movement ontogeny: limited variation in wild hatched birds and greater individual variation in spring and winter released birds. We propose that constraints motivate individuals to conform toward an optimal phenotype with limited variability around it while the lack of constraints provides the ecological opportunity for hidden individual variation in ontogeny to be expressed. It is remarkable that despite the striking individual variation in the timing to increase movement, all captive-hatched individuals started their migrations at approximately the same time, and after reaching similar maximum displacement distances, supporting the idea of an internal clock triggering movement shortly before migration. To validate our interpretation, that the internal clock of birds effectively paces the ontogeny of movement and to rule out rearing or weather conditions as alternative drivers, we would need to move the release of captive-hatched birds to the same time window as the fledging of the wild hatched birds. We would expect similarly synchronized performance increases of summer released vultures, as found in wild birds because the experienced time constraints would be similar. Importantly, data from the same birds ahead of their second migration corroborate that the observed first year patterns can indeed be interpreted as learning of flight behaviour. In their second summer, vultures move consistently over longer daily distances (30–35 km), with no increase in distance over the summer, no differences between wild hatched or captive-raised vultures, and little individual variation (electronic supplementary material, analysis S3). Because processes of learning, motivation and physiology can be directly interpreted from performance curves, studying the ontogeny of animal behaviour in the wild with performance curves opens new avenues to test biological hypotheses related to alternative developmental strategies. Future studies should harness experimental datasets with full controls or natural experiments, such as species that exhibit partial migration in their first year of life [22], to disentangle effects of internal clocks and environmental conditions driving the ontogeny of movement behaviour.

Our study system was based on a captive breeding programme and highlighted systematic differences in the ontogeny of flight behaviour between wild and captive hatched Egyptian vultures. For animals bred in captivity and released into the wild, the establishment of ‘natural’ movement behaviour, which resembles the behaviour of wild conspecifics, is pivotal for finding resources and escaping hazards [48,49] and hence ultimately for their survival and the success of the captive breeding programme [50]. However, such establishment of natural movement behaviour is often difficult to achieve for reintroduced individuals or populations, for example because movement can be socially learned and culturally transmitted over generations [5,51,52]. Direct comparisons of movement performance between wild hatched and captively raised individuals in the same environment are lacking from the literature, despite such comparisons being vital for evaluating the success of captive breeding programmes and the opportunity to use such comparative data to study ecological questions [53], as seen in our study. In the case of our Egyptian vulture system, we show that captive hatched birds can establish movement in a similar fashion to wild birds, as seen in some individuals, but that most birds released well in advance of their first migration in fact delay movement, probably in response to an internal clock, the zeitgeber of migration in combination with weather conditions favouring long flight distances in the summer.

## 5. Conclusion

We here propose that developmental schedules in some species could be more flexible than we currently see expressed, which could increase population resilience to a changing climate and extreme weather events. In fact, behavioural flexibility and individual plasticity have been suggested as key mechanisms for successfully coping with and persisting under climate change [54–56]. Climate change is already altering the timing of animal migrations [57,58], for example through individual plasticity [59], resulting in shifts of time constraints on adult breeding phenology [60] but also on juvenile ontogeny, as we suggest here.

## Data Availability

Data and code to reproduce all statistical analyses are published under the Open Science Framework [61]. Supplementary material is available online [62].
